# Pre- and postoperative communication with cataract patients and their family

**Published:** 2026-07-22

**Authors:** Ebby Adekhera, Lily A Nyamai, Lila Raj Puri

**Affiliations:** 1 Nursing Officer: Sabatia Eye Hospital, Wodanga, Kenya.; 2 Lecturer: Department of Special Surgeries, Kenyatta University, Nairobi, Kenya.; 3 Medical Advisor, Asia: The Fred Hollows Foundation, London, UK.

**Clear, compassionate patient education can improve safety, reduce anxiety, and support follow-up.**

**Figure:**
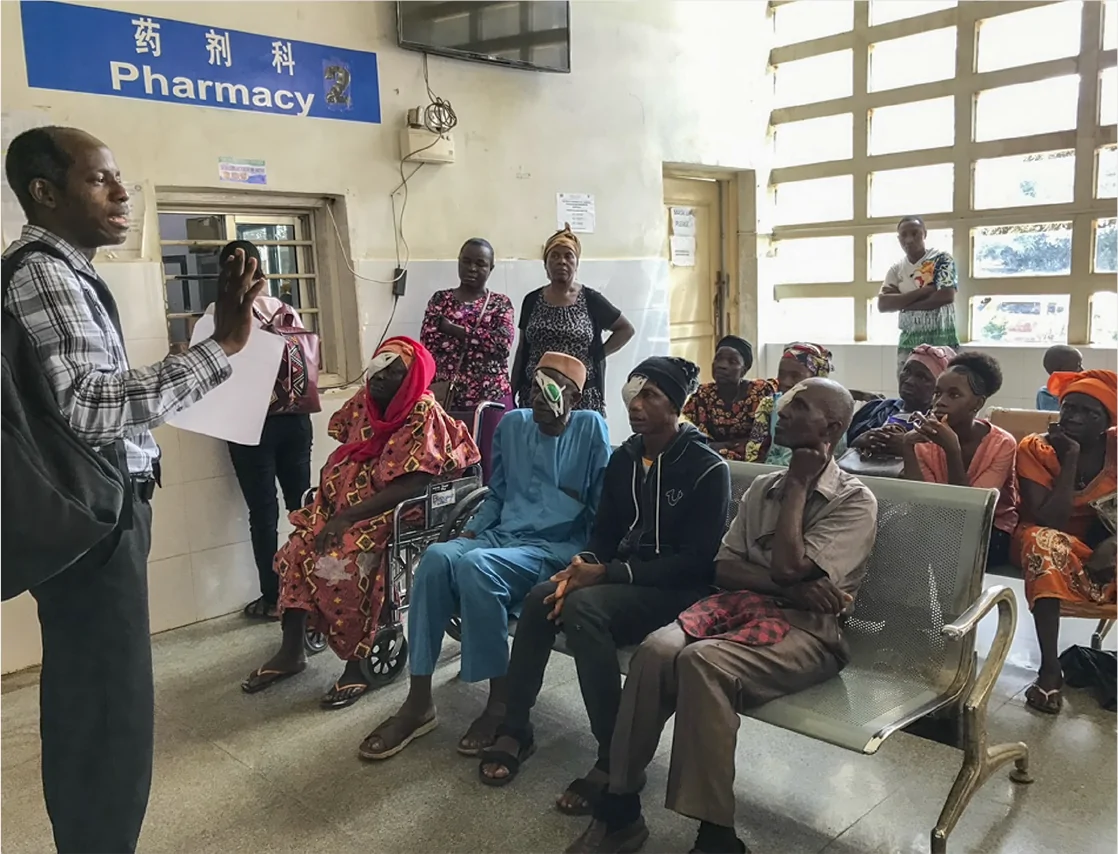
Postoperative group counselling of patients done prior to eye dressing removal. SIERRA LEONE

© HAMID KAMARA CC BY-NC-SA 4.0

With the huge backlog in cataract surgery in low- and middle-income countries worldwide, there is often a lot of pressure on eye care workers to get cataract patients through the system as quickly and efficiently as possible.

However, there is a compelling case for taking time to communicate clearly and compassionately with cataract patients and their family members before and after surgery – for example, encouraging them to ask questions, and confirming their understanding of what you have explained. If the patient doesn’t feel respected, they will not have a good experience – and will be less likely to encourage friends and family members to come forward. Worse, they may not feel confident to ask questions or share important information – or come back for follow-up visits – which may have devastating clinical implications. Our patients are human beings. It can be helpful to ask ourselves if we would be happy for our own parent or family member to receive the quality of care we provide to others.

## Before surgery

Give patients and their families advice and counselling about the operation, including what happens before, during and afterwards. They should then sign a consent form.

It is our responsibility to ensure that the patient understands – in straightforward terms – what is going to happen and what this means for them and their eye health. Take time to address any fears, doubts, and myths about cataract surgery.

It is important to ensure that patients and their families have realistic expectations about their vision after surgery. Help them to understand the risks and benefits, including the potential effect of different complications on their eyesight. A disappointed patient is not a good advertisement for our service.

Patients have a vital role to play in alerting the eye team to potential postoperative complications at an early stage. Before surgery, explain to patients how their eyes will look and feel after a successful operation, including what level of discomfort is normal during the recovery stage. Encourage them to speak with an eye team member if they experience anything that concerns them. This can be repeated after surgery.

*Adapted from Adekhera E, Nyamai LA, Astbury N. Pre- and postoperative communication with cataract patients, volume 29 issue 94, 2016. Substantially revised.* bit.ly/cehj433

**Table 1:** What advice to give to patients during follow-up appointments.

Follow-up stage	Day 1 (Immediate postoperative visit)	Week 1 (Early healing phase)	Week 4–6 (Final assessment, refraction, rehabilitation)
What advice to give	**Explain** how to care for their eye (what they must do, and what they must avoid) **Explain** the importance of instilling their eye drops (antibiotics and steroids) **Show** them how to instil eye drops safely **Explain** what normal healing will look and feel like, and how to manage their symptoms (mild pain & irritation) **Explain** what the danger signs are and the importance of getting help immediately – and how to do that	**Explain** how to gradually reduce the steroids as directed **Remind** them to complete the full course of antibiotics **Remind** them to avoid strenuous activity **Advise** that they can now wash the face/eye with clean, safe water. However, they must avoid swimming in untreated water.	**Perform refraction** if possible, or refer for refraction if needed **Advise** patients they can resume normal lifestyle **Advise** patients to watch for any gradual decline in vision (possible posterior capsule opacification) and to come back if this happens

It is helpful to have a team member who speaks the patient’s language. This can help to increase their understanding of any information being shared and boost their trust in the eye service. Ask questions to confirm that the patient understands everything they have been told.

If operations take place in an outreach location, counselling may be in a group setting. This may reduce individualised care but provides an opportunity for patients to share experiences and coping strategies, which can reduce anxiety and improve compliance.

## After surgery

After the operation, give patients and family members verbal and written information about the following:

- How to look after their operated eye when they are back at home.
- When to come back for a follow-up appointment: where to go, at what time, and how to reschedule if needed.
- How the eye will look and feel after surgery – i.e., what is normal and expected.
- What warning signs or red flags to look out for: worsening sight, increasing pain, redness, swelling, or discharge (see panel) – and how to get in touch with the eye clinic or their surgeon if they have these signs or symptoms.

## At discharge

Tell patients to bring all their eye drops to their follow-up appointments. Check that they have all of the following:

- Instruction sheet to take home
- Clinic contact details
- Eye shield (if available)
- Eye drops and instructions for storage and use
- Painkillers to use at home
- A follow-up appointment date and time.

**Taking care of the eye after cataract surgery**

Even if you feel well after surgery, be aware that you have had a major operation.

**What to do**

- If you are feeling uncomfortable, take your prescribed pain medication and wear sunglasses during the day to help with light sensitivity.
- Clean your eyelids gently morning and evening with a clean, moist cloth (avoid pressure on the eyeball). Avoid water entering the eye.
- Protect the operated eye for the first week: wear an eye shield at night, and sunglasses or spectacles during the day.
- Instil prescribed eye drops exactly as instructed.
- Resume your normal diet and any regular medications.
- Attend all follow-up appointments without fail.
- You may wash your hair after 1 day (avoid anything entering the eye).
- Take it easy for at least 2 weeks. You can resume sexual activity when comfortable.

**What to avoid**

- Don’t stop or restart any glaucoma or other regular eye medications unless specifically told to do so.
- Don’t use cotton wool near your eyes (the fibres can cause infection).
- Avoid sleeping on the operated side.
- Don’t lift heavy weights (>5 kg) for at least 2 weeks.
- Don’t wear eye make-up for at least 1 week.
- Avoid swimming for 2 weeks.

**Normal signs and symptoms after cataract surgery**

**How does it feel?**

- Mild discomfort, scratchy or gritty sensation (you can take the prescribed pain medication)
- Slight redness or watering
- Sensitivity to light
- Mild blurred vision that gradually improves
- Feeling of a ‘foreign body’ in the eye

**What does it look like?**

The following are normal:

- Slightly red or bloodshot eye
- Mild swelling of the eyelids
- Small red patch on the white of the eye (sometimes)

**Figure:**
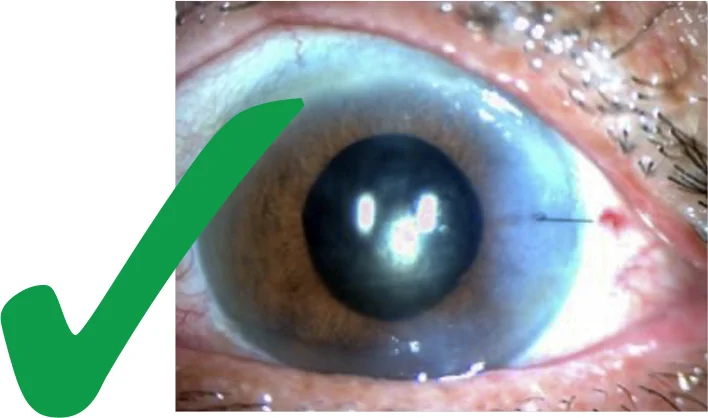
Normal appearance of the eye after surgery (no complications)

© ICEH CC BY-NC-SA 4.0

**Danger signs: Come back to the clinic immediately if you notice any of these**

Some discomfort, redness, or watering of the eye is normal, but it should get better over time. Come back immediately if the appearance of the eye, pain/discomfort, or your eyesight gets worse instead of better.

**Pain/discomfort**

Come back if **any** of the below are true:

- You have pain in your eye that is very strong, or getting stronger.
- You have a strong headache that is getting worse, especially if you feel sick or want to vomit.
- Bright light hurts your eye, or light is starting to hurt more than before.

**Appearance of the eye**

Ask someone to look at your eye if you are unsure, or if it is difficult to see clearly.

Come back if **any** of the below are true.

- The white of your eye becomes more red, or the redness is spreading (see photo **A**, below).
- The clear part at the front of your eye looks cloudy or hazy (photo **B**).
- There is white or yellow matter inside the front of the eye (photo **C**).
- You can see blood collecting inside the front of the eye (photo **D**).

**Your eyesight**

**Figure:**
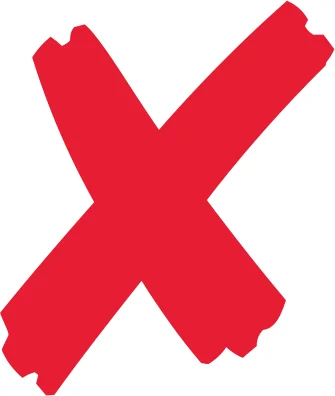

Come back if **any** of the below are true.

- Your sight is getting worse, or things look more blurry than before.
- You suddenly see a dark shadow, a dark patch, or a curtain coming across your sight.

**Figure:**
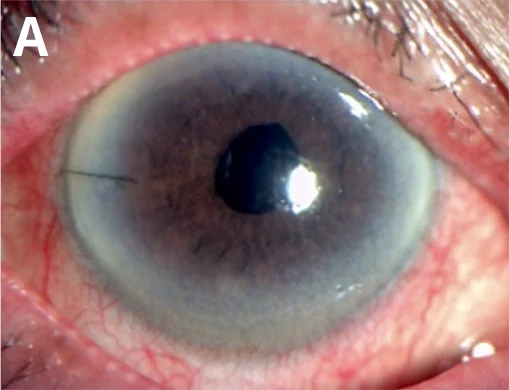
The white of the eye becomes more red

© LILA RAJ PURI CC BY-NC-SA 4.0

**Figure:**
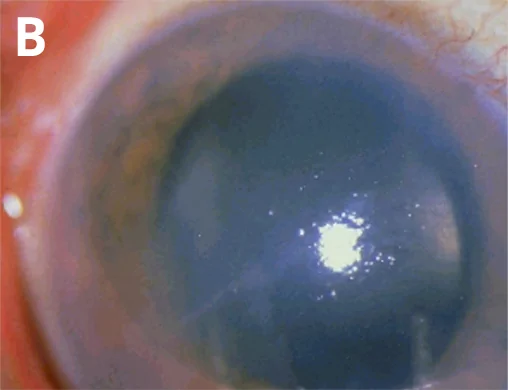
The clear part at the front of your eye looks cloudy or hazy

© LILA RAJ PURI CC BY-NC-SA 4.0

**Figure:**
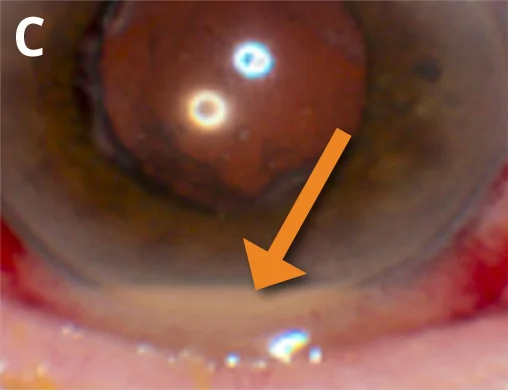
There is white or yellow matter inside the front of the eye

© LILA RAJ PURI CC BY-NC-SA 4.0

**Figure:**
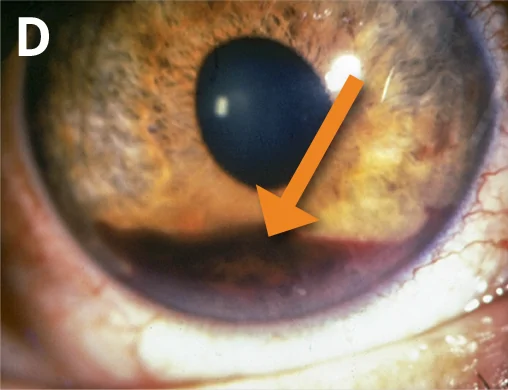
Blood collecting inside the front of the eye

© ALLEN FOSTER CC BY-NC-SA 4.0

